# Draft Genome Sequence of Lactobacillus plantarum Lp90 Isolated from Wine

**DOI:** 10.1128/genomeA.00097-15

**Published:** 2015-03-12

**Authors:** Antonella Lamontanara, Graziano Caggianiello, Luigi Orrù, Vittorio Capozzi, Vania Michelotti, Jumamurat R. Bayjanov, Bernadet Renckens, Sacha A. F. T. van Hijum, Luigi Cattivelli, Giuseppe Spano

**Affiliations:** a Consiglio Per la Ricerca e Sperimentazione in Agricoltura (CRA) Genomics Research Centre, Fiorenzuola d’Arda, Italy; b Department of Agriculture, Food and Environment Sciences, University of Foggia, Foggia, Italy; c Center for Molecular and Biomolecular Informatics, Radboud Institute for Molecular Life Sciences, Radboudumc, Nijmegen, The Netherlands; d NIZO food research, Ede, The Netherlands

## Abstract

Here, we describe the draft genome sequence and annotation of Lactobacillus plantarum strain Lp90, the first sequenced genome of a L. plantarum strain isolated from wine. This strain has a noticeable ropy phenotype and showed potential probiotic properties. The genome consists of 3,324,076 bp (33 contigs) and contains 3,155 protein coding genes, 34 pseudogenes, and 84 RNA genes.

## GENOME ANNOUNCEMENT

Lactobacillus plantarum is a bacterial species found in many different ecological niches such as vegetables, meat, fish, and dairy products as well as in the gastrointestinal tract (1). L. plantarum is one of the dominant bacteria in fermented foods such as sauerkraut, pickles, olives, wine, sourdough, and kimchi (1). Some strains are claimed to provide a health benefit and are marketed as probiotics (2). Here, we report the genome sequence of L. plantarum strain Lp90, a strain isolated from “Nero di Troia,” a typical Apulian (South of Italy) wine and in particular, from the same *terroir* of six Oenococcus oeni strains whose genomes have been recently sequenced (3, 4). This strain is characterized by a distinctive ropy phenotype, which was ascribed to its capacity to overproduce exopolysaccharides (EPS) (5), and by potential probiotic proprieties (unpublished results). This strain was already characterized in a previous study on phenotypic and genomic diversity of L. plantarum strains isolated from various environmental niches (6) and in three studies dedicated to Lp90 genes coding for small heat shock proteins (7–9). It is the first L. plantarum genome sequenced coming from a strain of wine origin. Two micrograms of genomic DNA was subjected to library preparation using the TruSeq DNA sample prep kit FC-121-1001 according to the manufacturer’s instructions. Whole-genome sequencing of Lp90 was performed using the Illumina GAIIx platform.

Prior to assembly, raw reads were filtered using the PRINSEQ v0.20.3 software (10). After filtering, a total of 16,574,199 paired-end reads ranging from 75 to 115 bp in length were obtained. The genome sequence was *de novo* assembled using the Ray v2.2.0 assembly program (11) with default parameters and using a Kmer size of 71. The assembly resulted in 33 contigs with an *N*_50_ length of 207,479 bp. The size of the shortest contig was 354 bp, while the length of the longest contig was 489,345 bp. The genome is 3,324,076 bp long with a GC content of 44.32%. Genome annotation was performed using the Rapid Annotation using Subsystem Technology (RAST) server (12). Functional annotations were refined by aligning the protein sequences to the Cluster of Orthologous Groups (COG) database (13) using BLASTp and by using the functionality of InterProScan v5.0 in Blast2GO (14) searching for matches against the PRINTS (v42.0), Pfam (v27.0), and TIGRFAMs (v13.0) databases. The TMHMM (v2.0) and Phobius (v1.01) prediction search tools were used, respectively, to predict transmembrane domains and the presence of signal peptides. Of the 3,273 predicted genes, 3,155 were protein-coding genes, 34 were identified to be pseudogenes, while 84 were RNA-coding genes (70 tRNAs and 14 rRNAs).

### Nucleotide sequence accession number.

The whole-genome shotgun project has been deposited at DDBJ/EMBL/GenBank under the accession number JIBX00000000.
